# Association of the C-reactive protein–triglyceride glucose index with albuminuria and macroalbuminuria: A population-based cross-sectional analysis

**DOI:** 10.1097/MD.0000000000049381

**Published:** 2026-06-19

**Authors:** Yanhua Huang, Hujiang Li, Yang Liu

**Affiliations:** a Chongqing University Three Gorges Hospital, Chongqing, China.

**Keywords:** a cross-sectional study, albuminuria, C-reactive protein–triglyceride glucose index, insulin resistance, macroalbuminuria

## Abstract

The C-reactive protein–triglyceride glucose index (CTI) is a novel marker that integrates systemic inflammation and insulin resistance, both of which are associated with renal injury. However, population-based data on its associations with albuminuria and macroalbuminuria remain limited. This cross-sectional study analyzed data from 15,163 US adults in the National Health and Nutrition Examination Survey 1999 to 2010. CTI was calculated as follows: 0.412 × ln(C-reactive protein [mg/dL]) + ln([triglycerides (mg/dL) × fasting glucose (mg/dL)]/2). Albuminuria and macroalbuminuria were defined as urinary albumin-to-creatinine ratio ≥30 mg/g and ≥300 mg/g, respectively. Multivariable logistic regression, adjusted for confounders, assessed the associations. Smooth curve fitting and threshold effect analysis revealed nonlinear associations. Subgroup and interaction analyses examined consistency. Receiver operating characteristic curves and area under the curve compared CTI’s predictive performance with other triglyceride-glucose (TyG)-related indices. The prevalence of albuminuria and macroalbuminuria among participants was 12.46% and 1.92%, respectively. Higher CTI was associated with increased risk (albuminuria: odds ratio 1.22, 95% confidence interval: 1.14–1.31; macroalbuminuria: odds ratio 1.43, 95% confidence interval: 1.22–1.68). Nonlinear analysis showed a U-shaped curve with an inflection point at CTI ≈ 8.00. The positive associations remained robust across all subgroups (age, sex, body mass index, hypertension, diabetes, physical activity, estimated glomerular filtration rate). CTI outperformed other TyG indices in receiver operating characteristic/area under the curve for predicting both outcomes. This cross-sectional analysis demonstrates a U-shaped association of CTI with albuminuria and macroalbuminuria, offering superior risk identification compared with traditional TyG markers.

## 1. Introduction

Epidemiological data indicate that approximately 15% to 20% of the global population has chronic kidney disease (CKD).^[1]^ Elevated urinary albumin excretion is an established early marker of renal dysfunction, crucial for identifying individuals at risk of CKD.^[2,3]^ A urinary albumin-to-creatinine ratio (ACR) >30 mg/g indicates elevated urinary albumin. In comparison, an ACR >300 mg/g (macroalbuminuria) suggests significant proteinuria, strongly associated with an increased risk of kidney failure.^[4]^ Extensive epidemiological studies confirm that albuminuria is an independent risk factor for both cardiovascular events and all-cause mortality.^[5]^

Recent studies recognize the triglyceride-glucose (TyG) index as a biomarker for evaluating insulin resistance (IR), linked to metabolic disorders and renal impairment.^[6–8]^ The TyG index, derived from fasting triglyceride (TG) and glucose levels, shows promise as a predictor of type 2 diabetes mellitus, cardiovascular events, and albuminuria.^[9,10]^ A Chinese study confirms that an elevated TyG is associated with albuminuria, with higher values linked to an increased risk of renal injury, particularly in individuals with poor glycemic control.^[11]^ Inflammation plays a critical role in proteinuria development. This inflammatory environment promotes endothelial dysfunction, podocyte injury, and systemic hypertension, contributing to an elevated risk of albuminuria and progression to CKD.^[12–14]^ The C-reactive protein–triglyceride glucose index (CTI), proposed by Ruan et al, integrates C-reactive protein (CRP) and TyG indices to assess inflammation and IR.^[15]^ It is strongly associated with depression, erectile dysfunction, endometriosis, and increased cancer mortality risk in the general population.^[16–19]^ Although prior studies have confirmed the TyG index’s association with proteinuria, the relationship between CTI and proteinuria, particularly macroalbuminuria, remains underexplored. Thus, we aimed to investigate CTI’s association with albuminuria and macroalbuminuria using a large National Health and Nutrition Examination Survey (NHANES) cohort to provide robust evidence.

## 2. Methods

### 2.1. Data selection and study design

The NHANES, conducted by the National Center for Health Statistics (NCHS) under the Centers for Disease Control and Prevention, is a cross-sectional study aimed at gathering health and nutritional information from a nationally representative sample of US households. Data were collected using a multistage probability sampling method, incorporating household interviews, physical examinations at mobile centers, and laboratory tests. This study was based on publicly available data from the NHANES, which is conducted by the NCHS. NHANES was approved by the NCHS Research Ethics Review Board, and all participants provided informed consent. The survey and data collection procedures were conducted in accordance with the principles of the Declaration of Helsinki. As this study involves secondary analysis of anonymized data, additional ethics approval was not required.

**Figure 1.**
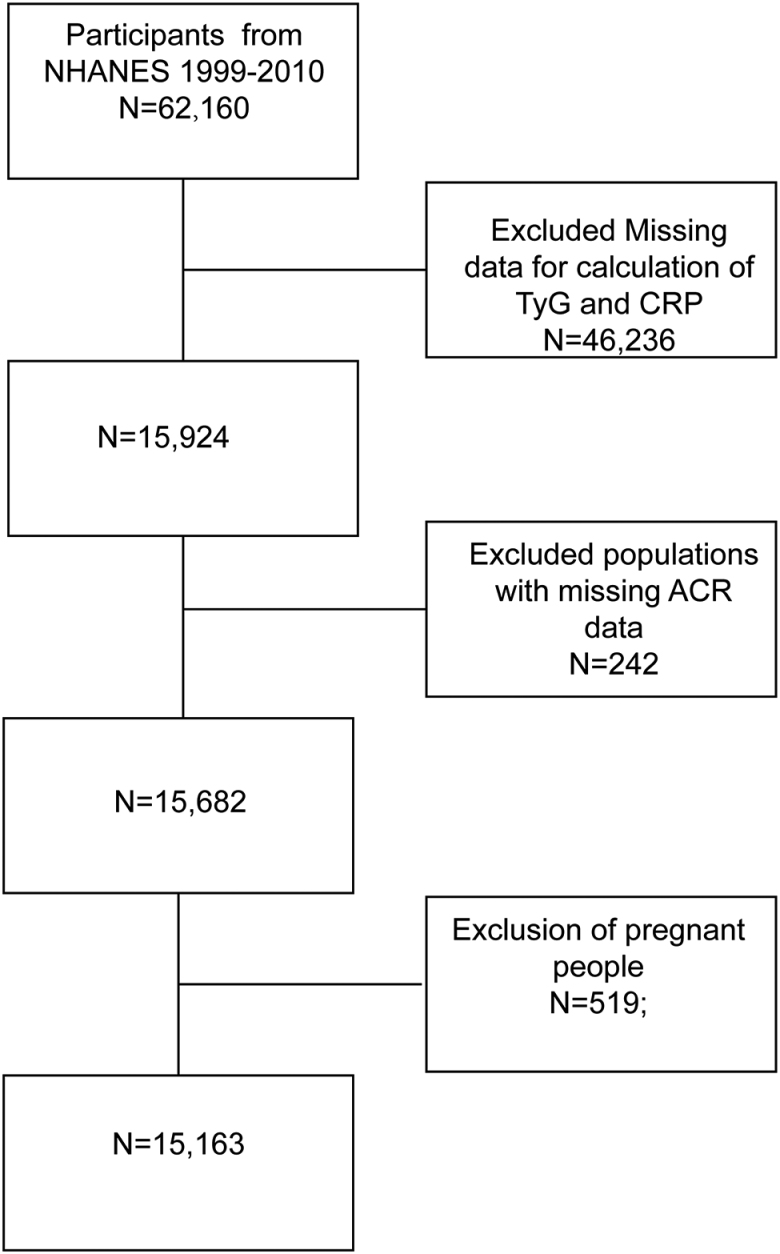
Flowchart of study participants. ACR, albumin-to-creatinine ratio, CRP = C-reactive protein, CTI = C-reactive protein–triglyceride glucose index, NHANES = National Health and Nutritional Examination Surveys, TyG = triglyceride-glucose.

We initially identified a total raw population of 62,160 participants from the general households surveyed in the NHANES 1999 to 2010 dataset, without applying any primary clinical diagnosis restrictions at this initial stage. We excluded 46,236 participants lacking fasting blood glucose, TG, or CRP values required for the CTI calculation. In addition, we excluded 242 participants with missing urinary ACR data. To ensure result reliability, we further excluded 519 pregnant participants. The full data screening process is detailed in Figure 1. The data used in this study were obtained from the NHANES, a publicly available dataset maintained by the Centers for Disease Control and Prevention. The data can be accessed at https://www.cdc.gov/nchs/nhanes/.

### 2.2. The definition of CTI

CTI was calculated using biochemical data from blood samples, including CRP, TGs, and fasting glucose, with the latter 2 reflecting IR. After an 8-hour fast, blood samples were collected and analyzed at an NCHS-certified laboratory. CTI was calculated as follows: CTI = 0.412 × ln(CRP [mg/dL]) + ln([TGs (mg/dL) × fasting glucose (mg/dL)]/2). Initially, CTI was analyzed as a continuous variable. To further explore potential nonlinear trends and clinical risk stratification, CTI was subsequently categorized into tertiles. Specifically, all participants were ranked in ascending order based on their calculated CTI scores and then divided into 3 equally sized, adjacent groups: tertile 1 (T1, lowest CTI values, representing the reference group), tertile 2 (T2, moderate CTI values), and tertile 3 (T3, highest CTI values), with each group accounting for approximately one-third of the total study population (N ≈ 5054 per group). Detailed information on analyzers and methods is available on the NHANES website (https://www.cdc.gov/nchs/nhanes/).

### 2.3. The definition of albuminuria and macroalbuminuria

Blood and urine samples from NHANES participants were collected at standardized mobile examination centers. Urinary albumin and creatinine were measured using solid-phase fluorescence immunoassay and modified Jaffe kinetic methods, respectively, from a single-spot urine sample. The urinary ACR was calculated as the urinary albumin concentration (mg) divided by the urinary creatinine concentration (g). Albuminuria was defined as an ACR ≥30 mg/g, and macroalbuminuria as an ACR ≥300 mg/g.

### 2.4. The covariates

Covariates included age (<60, ≥60 years), sex, race, education, marital status, poverty-to-income ratio, serum uric acid, low glomerular filtration rate (yes/no), body mass index (BMI), physical activity (vigorous or moderate), serum albumin, smoking status, alcohol consumption, diabetes mellitus, TGs, low-density lipoprotein cholesterol, alanine aminotransferase, and aspartate aminotransferase. Low glomerular filtration rate was defined as an estimated glomerular filtration rate (eGFR) <60 mL/min/1.73 m^2^, calculated using the Chronic Kidney Disease Epidemiology Collaboration (CKD-EPI) equation.^[1]^ Physical activity was categorized as vigorous (yes/no) or moderate (yes/no). Current smokers were defined as participants who had smoked ≥100 cigarettes in their lifetime and were smoking at the time of the survey. Former smokers were defined as participants who had smoked ≥100 cigarettes in their lifetime but had quit by the time of the survey. Nonsmokers were defined as participants who had smoked <100 cigarettes in their lifetime. Nondrinkers were participants who had consumed <12 alcoholic beverages in their lifetime or any single year. Former drinkers were participants who had consumed ≥12 alcoholic beverages in their lifetime or any single year but had abstained in the past 12 months. Current drinkers were participants who had consumed ≥12 alcoholic beverages in their lifetime or any single year and at least one in the past 12 months. Participants were classified as diabetic if they had physician-diagnosed diabetes, fasting blood glucose ≥7.0 mmol/L, glycosylated hemoglobin ≥6.5%, or a 75-g oral glucose tolerance test result indicative of diabetes. Hypertension was defined as systolic blood pressure ≥130 mm Hg, diastolic blood pressure ≥80 mm Hg, physician-diagnosed high blood pressure, or current use of antihypertensive medication.

### 2.5. Statistical methods

For baseline characteristics, group comparisons were performed using one-way analysis of variance for normally distributed continuous variables and the Kruskal–Wallis test for skewed continuous variables. The chi-square test was applied to compare categorical variables.

Multiple logistic regression models examined the associations of CTI with albuminuria and macroalbuminuria. Three models were applied: model 1 (unadjusted), model 2 (adjusted for age, sex, and race), and model 3 (adjusted for age, sex, race, BMI, education, marital status, poverty-to-income ratio, low eGFR, serum uric acid, serum albumin, TG, low-density lipoprotein cholesterol, diabetes mellitus, alcohol consumption, physical activity, smoking status, alanine aminotransferase, and aspartate aminotransferase). We further used smoothed curve fitting and threshold effect analysis to investigate the nonlinear associations between CTI and albuminuria and macroalbuminuria. Subgroup analyses assessed the stability of these associations across subgroups. Receiver operating characteristic (ROC) analysis and area under the curve (AUC) comparisons evaluated the performance of CTI and other TyG-derived indices in detecting albuminuria and macroalbuminuria. Missing values were addressed using multiple imputations. Analyses were conducted using R (version 3.4.3, R Foundation, http://www.r-project.org) and EmpowerStats (X&Y Solutions, Inc., Boston, http://www.empowerstats.com). Statistical significance was defined as *P* < .05.

### 2.6. Use of generative AI for language and grammar enhancement

During the preparation of this manuscript, we utilized the generative AI tool Kimi to assist in the refinement of language style and grammar. The tool was employed exclusively to polish the manuscript’s English expression and improve grammatical accuracy, without altering the scientific content or introducing new material.

## 3. Results

### 3.1. General characteristics of the study population

The study included 15,163 participants, with 1890 (12.46%) classified as having albuminuria and 291 (1.92%) as having macroalbuminuria. When grouped into tertiles, higher CTI levels were associated with a progressive increase in the prevalence of albuminuria and macroalbuminuria. Higher CTI levels were also associated with increased BMI and a higher prevalence of hypertension and diabetes mellitus (*P* < .001). Demographic characteristics are detailed in Table 1.

**Table 1:** Baseline characteristics of NHANES participants, 1999 to 2010 – categorized according to CTI tertiles.

Variables	Tertiles of CTI			*P* value
	T1	T2	T3	
N	5054	5054	5055	
UA (mg/dL)	4.88 ± 1.17	5.44 ± 1.34	5.87 ± 1.47	<.001
ALB (g/L)	44.11 ± 3.19	42.86 ± 3.29	41.67 ± 3.48	<.001
LDL-C (mmol/L)	2.47 ± 0.76	2.97 ± 0.90	3.13 ± 0.96	<.001
TG (mmol/L)	0.77 ± 0.28	1.23 ± 0.45	2.30 ± 1.71	<.001
ALT (U/L)	19.88 ± 10.77	25.75 ± 37.50	28.44 ± 22.34	<.001
AST (U/L)	24.16 ± 10.51	26.30 ± 33.61	26.60 ± 18.09	<.001
CTI	6.64 ± 0.48	7.80 ± 0.26	8.92 ± 0.57	<.001
Gender, n (%)				.001
** **Male	2581 (51.07%)	2673 (52.89%)	2487 (49.20%)	
** **Female	2473 (48.93%)	2381 (47.11%)	2568 (50.80%)	
Age, n (%)				<.001
** **<60	4618 (91.37%)	3598 (71.19%)	2971 (58.77%)	
** **≥60	436 (8.63%)	1456 (28.81%)	2084 (41.23%)	
Race, n (%)				<.001
** **Mexican American	1104 (21.84%)	1157 (22.89%)	1316 (26.03%)	
** **Other Hispanic	315 (6.23%)	386 (7.64%)	392 (7.75%)	
** **Non-Hispanic White	1848 (36.57%)	2279 (45.09%)	2356 (46.61%)	
** **Non-Hispanic Black	1519 (30.06%)	1030 (20.38%)	816 (16.14%)	
** **Other Race	268 (5.30%)	202 (4.00%)	175 (3.46%)	
Education, n (%)				<.001
** **Under high school	528 (10.45%)	1187 (23.49%)	1724 (34.10%)	
** **High school or equivalent	506 (10.01%)	982 (19.43%)	1163 (23.01%)	
** **College graduate or above	4020 (79.54%)	2885 (57.08%)	2168 (42.89%)	
Marital status, n (%)				<.001
** **Married or living with partner	2699 (53.40%)	3049 (60.33%)	3103 (61.38%)	
** **Living alone	2355 (46.60%)	2005 (39.67%)	1952 (38.62%)	
PIR				<.001
** **<1.3	1569 (31.04%)	1421 (28.12%)	1581 (31.28%)	
** ≥**1.3, <3.5	2099 (41.53%)	2214 (43.81%)	2257 (44.65%)	
** ≥**3.5	1386 (27.42%)	1419 (28.08%)	1217 (24.08%)	
Low eGFR				<.001
** **No	4972 (98.38%)	4712 (93.23%)	4514 (89.30%)	
** **Yes	82 (1.62%)	342 (6.77%)	541 (10.70%)	
Albuminuria				<.001
** **No	4579 (90.60%)	4548 (89.99%)	4146 (82.02%)	
** **Yes	475 (9.40%)	506 (10.01%)	909 (17.98%)	
Smoke, n (%)				<.001
** **Current smokers	481 (9.52%)	877 (17.35%)	1098 (21.72%)	
** **Nonsmokers	4105 (81.22%)	3104 (61.42%)	2554 (50.52%)	
** **Former smokers	468 (9.26%)	1073 (21.23%)	1403 (27.75%)	
Diabetes, n (%)				<.001
** **No	4945 (97.84%)	4569 (90.40%)	3579 (70.80%)	
** **Yes	109 (2.16%)	485 (9.60%)	1476 (29.20%)	
BMI, n (%)				<.001
** **<25	3537 (69.98%)	1681 (33.26%)	739 (14.62%)	
** **≥25, <30	1111 (21.98%)	1883 (37.26%)	1717 (33.97%)	
** **≥30	406 (8.03%)	1490 (29.48%)	2599 (51.41%)	
Vigorous activity, n (%)				<.001
** **No	2194 (43.41%)	3101 (61.36%)	3788 (74.94%)	
** **Yes	2860 (56.59%)	1953 (38.64%)	1267 (25.06%)	
Moderate activity, n (%)				<.001
** **No	2441 (48.30%)	2689 (53.21%)	2977 (58.89%)	
** **Yes	2613 (51.70%)	2365 (46.79%)	2078 (41.11%)	
Drink, n (%)				<.001
** **Current drinkers	4468 (88.41%)	3863 (76.43%)	3267 (64.63%)	
** **Nondrinkers	271 (5.36%)	461 (9.12%)	671 (13.27%)	
** **Former drinkers	315 (6.23%)	730 (14.44%)	1117 (22.10%)	
Hypertension				<.001
** **No	4058 (80.29%)	2827 (55.94%)	1848 (36.56%)	
** **Yes	996 (19.71%)	2227 (44.06%)	3207 (63.44%)	
Macroalbuminuria				<.001
** **No	5015 (99.23%)	4988 (98.69%)	4869 (96.32%)	
** **Yes	39 (0.77%)	66 (1.31%)	186 (3.68%)	

The baseline characteristics table presented continuous variables as means with standard deviation, while categorical variables were reported as proportions. Group comparisons were performed using one-way analysis of variance for normally distributed continuous variables and the Kruskal–Wallis test for skewed continuous variables. The chi-square test was applied to compare categorical variables.

ALT = alanine aminotransferase, AST = aspartate aminotransferase, BMI = body mass index, CTI = C-reactive protein–triglyceride glucose index, eGFR = estimated glomerular filtration rate, LDL-C = low-density lipoprotein cholesterol, PIR = poverty-to-income ratio, TG = triglycerides, UA = uric acid.

### 3.2. Relationship of CTI to albuminuria and macroalbuminuria

Multivariate logistic regression examined the associations of CTI with albuminuria and macroalbuminuria in model 1, model 2, and model 3. Table 2 presents significant positive associations between CTI and both albuminuria and macroalbuminuria across all models. In the fully adjusted model 3, each unit increase in CTI was associated with a 22% higher odds of albuminuria (odds ratio = 1.22, 95% confidence interval [95% CI]: 1.14–1.31). Each unit increase in CTI was associated with a 43% higher odds of macroalbuminuria (odds ratio = 1.43, 95% CI: 1.22–1.68). Continuous CTI values were categorized into tertiles. In model 3, the prevalence of albuminuria was 19% higher in the highest tertile (T3) than in the lowest tertile (T1; *P*-trend = .02). The prevalence of macroalbuminuria showed a consistent trend, with a 31% higher prevalence in T3 than in the lowest tertile (T1), although not statistically significant (*P* for trend = .1204).

**Table 2:** Association of CTI with albuminuria and macroalbuminuria in the population.

	Model 1, OR (95% CI)	Model 2, OR (95% CI)	Model 3, OR (95% CI)
Albuminuria	1.48 (1.41–1.55)	1.34 (1.27–1.41)	1.22 (1.14–1.31)
** **T1	Ref	Ref	Ref
** **T2	1.07 (0.94–1.22)	0.87 (0.76–0.99)	0.85 (0.73–1.00)
** **T3	2.11 (1.88–2.38)	1.52 (1.34–1.73)	1.19 (1.00–1.42)
** ***P* for trend	<.0001	<.0001	.02
Macroalbuminuria	2.22 (1.99–2.47)	2.11 (1.88–2.36)	1.43 (1.22–1.68)
** **T1	Ref	Ref	Ref
** **T2	1.70 (1.14–2.53)	1.43 (0.95–2.15)	0.96 (0.61–1.49)
** **T3	4.91 (3.47–6.95)	3.72 (2.58–5.37)	1.31 (0.83–2.09)
** ***P* for trend	<.0001	<.0001	.1204

Model 1: No covariates were adjusted.

Model 2: Adjusted for age, gender, and race/ethnicity.

Model 3: Age, sex, race, body mass index, education, marital status, PIR, low eGFR, serum uric acid, serum albumin, TG, LDL-C, diabetes mellitus, alcohol consumption, physical activity, smoking status, ALT, and AST were adjusted.

95% CI = 95% confidence interval, ALT = alanine aminotransferase, AST = aspartate aminotransferase, CTI = C-reactive protein–triglyceride glucose index, eGFR = estimated glomerular filtration rate, LDL-C = low-density lipoprotein cholesterol, OR = odds ratio, PIR = poverty-to-income ratio, TG = triglycerides.

**Figure 2.**
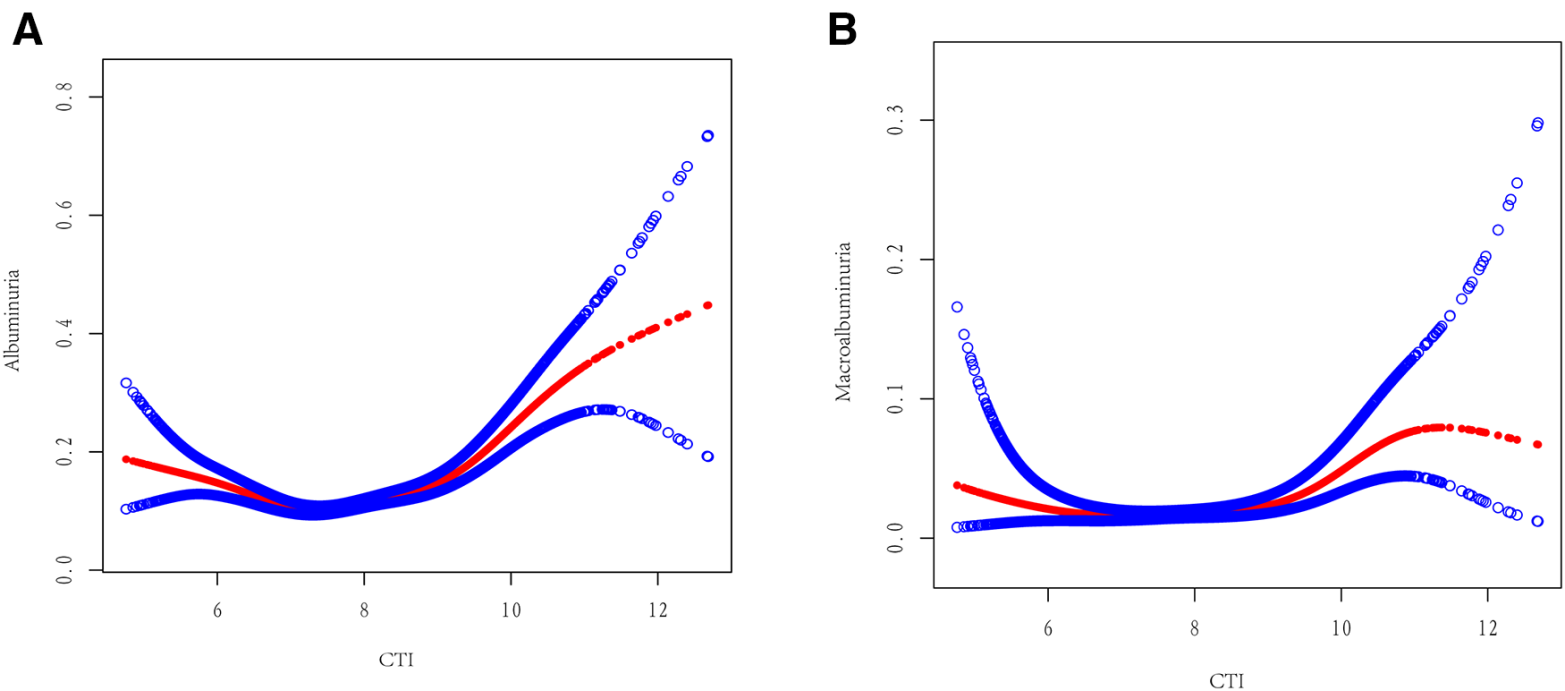
(A) Smooth curve fitting diagram of CTI and albuminuria in model 3. (B) Smooth curve fitting graph of CTI and macroalbuminuria in model 3. The red line represents albuminuria and macroalbuminuria prevalence, while the blue band indicates its 95% confidence interval. The X-axis denotes CTI (continuous variable), and the Y-axis denotes albuminuria and macroalbuminuria prevalence. Adjusted factors in model 3: age, sex, race, body mass index, education, marital status, PIR, low eGFR, serum uric acid, serum albumin, TG, LDL-C, diabetes mellitus, alcohol consumption, physical activity, smoking status, ALT, and AST. ALT = alanine aminotransferase, AST = aspartate aminotransferase, CTI = C-reactive protein–triglyceride glucose index, eGFR = estimated glomerular filtration rate, LDL-C = low-density lipoprotein cholesterol, PIR = poverty-to-income ratio, TG = triglycerides.

Figure 2 shows that smoothed curve fitting revealed a U-shaped nonlinear relationship between CTI and both albuminuria and macroalbuminuria. Threshold effect analysis (Table 3) identified a CTI cutoff of 8.00, with the prevalence of albuminuria and macroalbuminuria decreasing when CTI < 8.00 and increasing when CTI > 8.00.

**Table 3:** Threshold effects of CTI on albuminuria and macroalbuminuria analyzed using linear regression models.

	Adjusted OR (95% CI), *P* value
Albuminuria	
** **Fitting by the standard linear model	1.22 (1.14–1.31), <.0001
** **Fitting by the 2-piecewise linear model	
** **CTI	
** **Inflection point	8.00
** **CTI < 8.00	0.85 (0.76–0.95), .0039
** **CTI > 8.00	1.63 (1.47–1.80), <.0001
** **Log likelihood ratio	<0.001
Macroalbuminuria	
** **Fitting by the standard linear model	1.43 (1.22–1.68), <.0001
** **Fitting by the 2-piecewise linear model	
** **CTI	
** **Inflection point	8.00
** **CTI < 8.00	0.87 (0.62–1.21), .3970
** **CTI > 8.00	1.72 (1.42–2.08), .0011
** **Log likelihood ratio	0.001

Adjusted for sex, age, race, PIR, BMI, education level, marital status, diabetes, hypertension, triglycerides, low eGFR, low-density lipoprotein, alcohol consumption, smoking status, physical activity, ALT, AST, albumin, and uric acid.

95% CI = 95% confidence interval, ALT = alanine aminotransferase, AST = aspartate aminotransferase, BMI = body mass index, CTI = C-reactive protein–triglyceride glucose index, eGFR = estimated glomerular filtration rate, LDL-C = low-density lipoprotein cholesterol, OR = odds ratio, PIR = poverty-to-income ratio, TG = triglycerides.

### 3.3. The subgroup analysis

Subgroup analyses assessed whether the associations of CTI with albuminuria and macroalbuminuria were consistent across subgroups. Table 4 shows that the positive associations between CTI and both albuminuria and macroalbuminuria persisted across all subgroups, including age, sex, BMI, hypertension, diabetes mellitus, vigorous physical activity, moderate physical activity, and low eGFR. Detailed results are presented in Table 4.

**Table 4:** Subgroup analysis of the relationship between CTI and albuminuria and macroalbuminuria in model 3.

Character	OR (95% CI)	*P* value	*P* for interaction
**Albuminuria**			<.0001
BMI			
** **Below 25	1.02 (0.91, 1.15)	.6745	
** **25–29.9	1.37 (1.20, 1.57)	<.0001	
** **≥30	1.51 (1.33, 1.72)	<.0001	
Drink			<.0001
** **Current drinkers	1.12 (1.03, 1.21)	.0062	
** **Nondrinkers	1.44 (1.22, 1.71)	<.0001	
** **Former drinkers	1.50 (1.31, 1.71)	<.0001	
Hypertension			.0005
** **Yes	1.39 (1.27, 1.53)	<.0001	
** **No	1.07 (0.96, 1.20)	.2332	
Diabetes			<.0001
** **Yes	1.48 (1.33, 1.65)	<.0001	
** **No	1.09 (1.00, 1.19)	.0440	
Vigorous activity			<.0001
** **Yes	1.06 (0.96, 1.17)	.2676	
** **No	1.31 (1.21, 1.42)	<.0001	
Moderate activity			.0953
** **Yes	1.16 (1.06, 1.27)	.0012	
** **No	1.26 (1.16, 1.37)	<.0001	
Marital status			.6066
** **Married or living with partner	1.23 (1.13, 1.34)	<.0001	
** **Living alone	1.20 (1.10, 1.31)	<.0001	
Smoke			<.0001
** **Current smokers	1.66 (1.44, 1.91)	<.0001	
** **Nonsmokers	1.07 (0.98, 1.16)	.1154	
** **Former smokers	1.38 (1.22, 1.57)	<.0001	
Age			.1492
** **<60	1.18 (1.09, 1.28)	<.0001	
** ≥**60	1.29 (1.16, 1.43)	<.0001	
Gender			.0013
** **Male	1.40 (1.26, 1.55)	<.0001	
** **Female	1.11 (1.00, 1.22)	.0449	
Low eGFR			.0931
** **Yes	1.39 (1.17, 1.65)	.0002	
** **No	1.20 (1.11, 1.29)	<.0001	
**Macroalbuminuria**			
BMI			.4341
** **Below 25	1.52 (1.15, 2.00)	.0036	
** **25–29.9	1.25 (0.92, 1.69)	.1481	
** **≥30	1.63 (1.24, 2.15)	.0005	
Drink			.1690
** **Current drinkers	1.29 (1.07, 1.57)	.0086	
** **Nondrinkers	1.75 (1.23, 2.49)	.0018	
** **Former drinkers	1.59 (1.22, 2.05)	.0005	
Hypertension			.3319
** **Yes	1.53 (1.27, 1.84)	<0.0001	
** **No	1.26 (0.89, 1.78)	0.1975	
Diabetes			.0280
** **Yes	1.63 (1.34, 1.99)	<.0001	
** **No	1.19 (0.95, 1.50)	.1215	
Vigorous activity			.8305
** **Yes	1.40 (1.08, 1.81)	.0114	
** **No	1.44 (1.21, 1.71)	<.0001	
Moderate activity			.9433
** **Yes	1.42 (1.13, 1.79)	.0029	
** **No	1.43 (1.20, 1.71)	<.0001	
Marital status			.2014
** **Married or living with partner	1.33 (1.10, 1.61)	.0037	
** **Living alone	1.55 (1.26, 1.91)	<.0001	
Smoke			.5022
** **Current smokers	1.33 (1.01, 1.76)	.0393	
** **Nonsmokers	1.39 (1.14, 1.69)	.0013	
** **Former smokers	1.63 (1.24, 2.14)	.0005	
Age			.5570
** **<60	1.38 (1.14, 1.68)	.0012	
** **≥60	1.49 (1.20, 1.84)	.0003	
Gender			.4492
** **Male	1.53 (1.23, 1.91)	.0001	
** **Female	1.35 (1.06, 1.73)	.0164	
Low eGFR			.4213
** **Yes	1.31 (1.01, 1.70)	.0393	
** **No	1.47 (1.24, 1.76)	<.0001	

Model 3: age, sex, race, body mass index, education, marital status, PIR, low eGFR, serum uric acid, serum albumin, TG, LDL-C, diabetes mellitus, alcohol consumption, physical activity, smoking status, ALT, and AST were adjusted.

95% CI = 95% confidence interval, ALT = alanine aminotransferase, AST = aspartate aminotransferase, BMI = body mass index, CTI = C-reactive protein–triglyceride glucose index, eGFR = estimated glomerular filtration rate, LDL-C = low-density lipoprotein cholesterol, OR = odds ratio, PIR = poverty-to-income ratio, TG = triglycerides.

### 3.4. CTI is more capable of recognizing albuminuria and macroalbuminuria than other TyG-derived metrics

**Figure 3.**
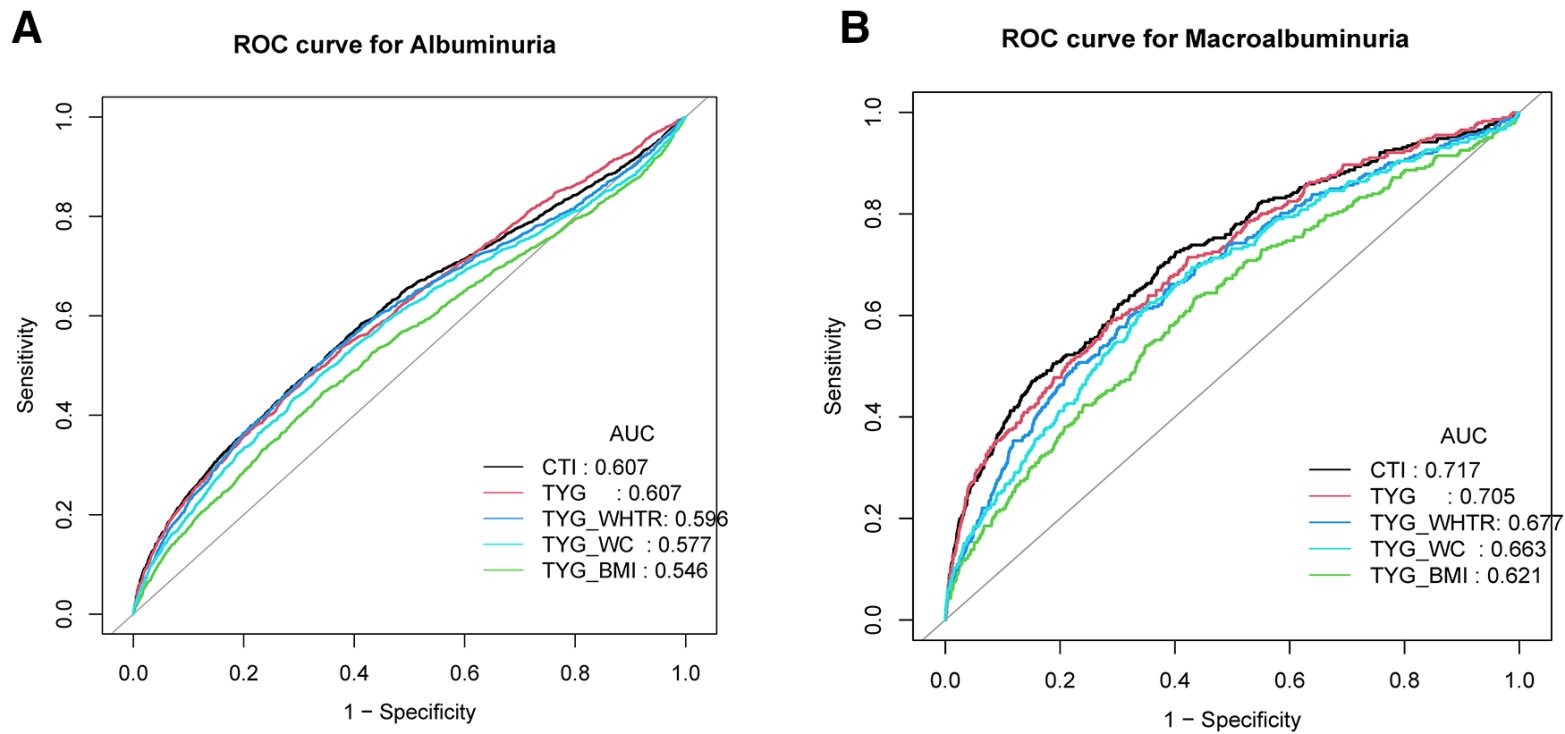
The CTI and other TyG-derived indicators (TyG, TyG-WHtR, TyG-WC, TyG-BMI) were employed to generate receiver operating characteristic curves and determine their respective area under the curve values for the detection of albuminuria and macroalbuminuria. (A) Five metrics were evaluated to assess their ability to identify albuminuria. (B) Five metrics were evaluated to assess their ability to identify macroalbuminuria. 95% CI = 95% confidence interval, AUC = area under the curve, CTI = C-reactive protein–triglyceride glucose index, TyG = triglyceride-glucose, TyG-BMI = triglyceride-glucose body mass index, TyG-WC = triglyceride-glucose waist circumference, TyG-WHtR = triglyceride-glucose waist-to-height ratio.

The AUC was calculated to compare the performance of CTI against TyG and TyG-derived indices (triglyceride-glucose waist-to-height ratio, triglyceride-glucose waist circumference, triglyceride-glucose body mass index [TyG-BMI]) in detecting albuminuria and macroalbuminuria. The formulas for TyG and TyG-derived indices can be found in the Supplementary Material, Supplemental Digital Content 1. Figure 3 presents ROC curves evaluating the performance of CTI and other indices in detecting albuminuria and macroalbuminuria. Table 5 lists AUC values (95% CI) for albuminuria detection, with CTI showing the highest AUC (0.6072), significantly outperforming TyG-BMI and triglyceride-glucose waist circumference (*P* < .05). For macroalbuminuria detection, CTI had the highest AUC (0.7165) among the indices. Table 5 provides details, including optimal thresholds, sensitivity, and specificity. For instance, the optimal CTI threshold for detecting macroalbuminuria was 8.0452 (specificity: 0.7099, sensitivity: 0.4338). These findings indicate that CTI outperforms other TyG-derived indices in detecting albuminuria and macroalbuminuria.

**Table 5:** Comparison of AUC values of CTI with other TyG and its derivatives markers.

Test	AUC	95% CI low	95% CI upp	Best threshold	Specificity	Sensitivity	*P* for different in AUC
Albuminuria							
** **CTI	0.6072	0.5925	0.6219	7.9761	0.5865	0.5852	reference
** **TyG	0.6065	0.5922	0.6208	8.8128	0.6905	0.4730	.8637
** **TyG-BMI	0.5458	0.5307	0.5610	253.2112	0.6558	0.4771	<.0001
** **TyG-WHtR	0.5955	0.5804	0.6107	5.1342	0.6348	0.5334	.0633
** **TyG-WC	0.5772	0.5619	0.5924	899.5706	0.7099	0.4338	<.0001
Macroalbuminuria							
** **CTI	0.7165	0.6843	0.7488	8.0452	0.5986	0.7251	reference
** **TyG	0.7049	0.6727	0.7371	8.8972	0.7173	0.5842	.1869
** **TyG-BMI	0.6211	0.5861	0.6561	239.4306	0.5665	0.6335	<.0001
** **TyG-WHtR	0.6767	0.6425	0.7110	858.6000	5.3141	0.6788	.0011
** **TyG-WC	0.6634	0.6292	0.6976	872.0181	0.6442	0.6250	<.0001

95% CI = 95% confidence interval, AUC = area under the curve, CTI = C-reactive protein–triglyceride glucose index, TyG = triglyceride-glucose, TyG-BMI = triglyceride-glucose body mass index, TyG-WC = triglyceride-glucose waist circumference, TyG-WHtR = triglyceride-glucose waist-to-height ratio.

## 4. Discussion

This large cross-sectional study using the NHANES 1999 to 2010 dataset identified a U-shaped association between CTI levels and both albuminuria and macroalbuminuria, with a threshold effect at CTI = 8.00. ROC analysis and AUC values suggest that CTI has better discriminatory ability than other TyG-derived indices for albuminuria and macroalbuminuria.

Our findings align with prior studies linking IR and inflammation to renal injury. For instance, the TyG index, a CTI component, is associated with albuminuria.^[9,20]^ However, this study identified a nonlinear relationship between CTI and albuminuria, incorporating CRP. We observed a lower prevalence of albuminuria and macroalbuminuria at CTI <8.00 and a higher prevalence at CTI >8.00. Our findings align with Shen et al, who reported U-shaped associations of triglyceride-glucose waist-to-height ratio and TyG-BMI with CKD and a J-shaped association of TyG with CKD.^[7]^ We extended this by examining the association of CTI with proteinuria, an early indicator of renal injury. Chen et al, in a longitudinal cohort study, also identified a U-shaped association between TyG-BMI and both all-cause and cardiovascular mortality in patients with CKD.^[21]^ Our study identified a U-shaped association between CTI and both albuminuria and macroalbuminuria, indicating complex effects of inflammation and IR on early renal injury.

The association between CTI levels and albuminuria and macroalbuminuria is complex, likely mediated by molecular pathways involving inflammation and IR. Insulin resistance activates pro-inflammatory pathways, contributing to renal injury. Elevated CTI reflects increased IR, promoting inflammatory cytokine release (e.g., interleukin-6, tumor necrosis factor-alpha), which drives chronic low-grade inflammation.^[22]^ Inflammation exacerbates renal endothelial dysfunction and disrupts glomerular hemodynamics. This disruption increases glomerular filtration barrier permeability, causing albumin leakage and albuminuria.^[23]^ Altered insulin signaling may also activate the renin-angiotensin-aldosterone system (RAAS). Insulin promotes vasodilation by stimulating endothelial nitric oxide production via the phosphatidylinositol 3-kinase pathway.^[24]^ In insulin-resistant states, the phosphatidylinositol 3-kinase pathway is impaired, while the mitogen-activated protein kinase pathway promotes vasoconstriction. Insulin resistance enhances RAAS activity, contributing to hypertension and renal injury.^[25]^ RAAS activation promotes glomerular hypertension and hyperfiltration, increasing proteinuria risk.^[26]^ Inflammation-induced oxidative stress impairs renal proximal tubular function, further contributing to albuminuria.^[27]^ Thus, the pathway from CTI to albuminuria is multifactorial, involving inflammation, oxidative stress, and hemodynamic alterations.

The U-shaped association between CTI and albuminuria indicates the dual effects of IR and inflammation on renal health. At lower CTI levels, an insulin-sensitive state supports normal renal function and reduces inflammation, protecting against renal injury. Conversely, at higher CTI levels, IR and elevated inflammation impair renal function, causing proteinuria. However, the U-shaped association suggests that overemphasizing IR may obscure protective mechanisms at mild CTI levels. Studies indicate that health indices and lifestyle factors, for example, sleep duration,^[28,29]^ also show U-shaped associations with albuminuria and CKD, suggesting that insufficient or excessive biological activity contributes to adverse renal outcomes. The U-shaped association is further supported by physiological mechanisms indicating a compensatory response. One possible explanation is that mild declines in insulin sensitivity may trigger compensatory adaptations that temporarily mitigate renal stress until these mechanisms are overwhelmed by more severe insulin resistance.

Unlike prior studies, we examined the association between CTI and proteinuria using albuminuria and macroalbuminuria as outcome variables, demonstrating result stability. However, several limitations should be acknowledged. First, the cross-sectional design prevents confirmation of a causal relationship between CTI and proteinuria. Second, sample selection bias was unavoidable, as only a subset of the original sample met the inclusion criteria. Third, the use of North American population-based data limits the generalizability of findings. Future studies should use longitudinal cohort data to establish causality and include diverse populations, such as Asian cohorts, for validation. Finally, molecular biology studies are needed to elucidate the mechanisms underlying the U-shaped association between CTI and proteinuria.

## 5. Conclusion

Using data from the analyzed NHANES cohort, our study identified a U-shaped association between CTI and the prevalence of albuminuria and macroalbuminuria. CTI reflects IR and chronic inflammation, key mechanisms in the onset and progression of proteinuria. Thus, CTI may serve as a clinical biomarker to support early prevention or intervention for proteinuria. However, further longitudinal studies are needed to validate these findings.

## Author contributions

**Data curation:** Yanhua Huang, Hujiang Li.

**Formal analysis:** Yanhua Huang.

**Investigation:** Yanhua Huang.

**Software:** Yanhua Huang.

**Methodology:** Hujiang Li, Yang Liu.

**Resources:** Hujiang Li.

**Conceptualization:** Yang Liu.

**Supervision:** Yang Liu.

**Validation:** Yang Liu.

**Writing – review & editing:** Yang Liu.

**Writing – original draft:** Yanhua Huang, Hujiang Li.


