# Supplementary material for: Association of the C-reactive protein–triglyceride glucose index with albuminuria and macroalbuminuria: A population-based cross-sectional analysis
Source: Medicine (Baltimore). 2026 Jun 19;105(25):e49381. doi: 10.1097/MD.0000000000049381 (PMC13286468; doi:10.1097/MD.0000000000049381)
Supplement: Supplementary file 1 [file medi-105-e49381-s001.docx]

**Calculation instructions for TyG and its derivatives**

TyG, TyG-WC, TyG-WHtR, and TyG-BMI were calculated according to the following formulas:

(1) TyG = ln [triglycerides (mg/dl) × glucose (mg/dl)/2];

(2) BMI = body mass (kg)/height^2^(m^2^);

(3) WHtR = waist circumference/height;

(4) TyG-WC = TyG × waist circumference; TyG-WHtR = TyG × WHtR; TyG-BMI = TyG × BMI.
